# Supplementary material for: Mechanical Control of Cell Migration by the Metastasis Suppressor Tetraspanin CD82/KAI1
Source: Cells. 2021 Jun 18;10(6):1545. doi: 10.3390/cells10061545 (PMC8234748; doi:10.3390/cells10061545)
Supplement: Supplementary file 1 [file cells-10-01545-s001.zip › cells-1192922-supplementary.pdf]

Supplementary information

10 movies:

Supplementary Movie S1: HB2 single cell migration. Persistent cell migration can be observed (Figure S1).

Supplementary Movie S2: HB2-CD82 single cell migration. Cell migration is impaired, cells turn round (Figure S1).

Supplementary Movie S3: MCF10a cells transfected with Si Ctl single cell migration: they express CD82 and turn in round. (Figure S2).

Supplementary Movie S4: MCF10a cells transfected with siCD82 single cell migration: persistent cell migration can be observed. (Figure S2)

Supplementary Movie S5: Talin-GFP dynamics in 1 (out of 10) HB2 migrating cell. Notice the sliding of talin-GFP spots, retrograde from the migration front (Figure S1).

Supplementary Movie S6: Talin-GFP dynamics in 1 (out of 10) HB2-CD82 migrating cell where talin-GFP clusters are mostly immobile (Figure S1).

Supplementary Movie S7: Talin-GFP dynamics in 1 (out of 5) MCF10 si Ctl. The cell shows large peripheral adhesion sites and are slowly moving (Figure S3).

Supplementary Movie S8: Talin-GFP dynamics in 2 (out of 5) MCF10a siCD82. Cells show small and dynamic adhesion sites and are moving faster than controls (Figure S3).

Supplementary Movie S9: Caveolin-GFP dynamics in 1 (out of 10) HB2 cells in iso (left) and after 5 min hypoosmotic (right) condition observed by TIRF microscopy.

Supplementary Movie S10: Caveolin-GFP dynamics in 1 (out of 10) HB2-CD82 cell in iso (left) and after 5 min hypoosmotic (right) condition observed by TIRF microscopy.

Figures:

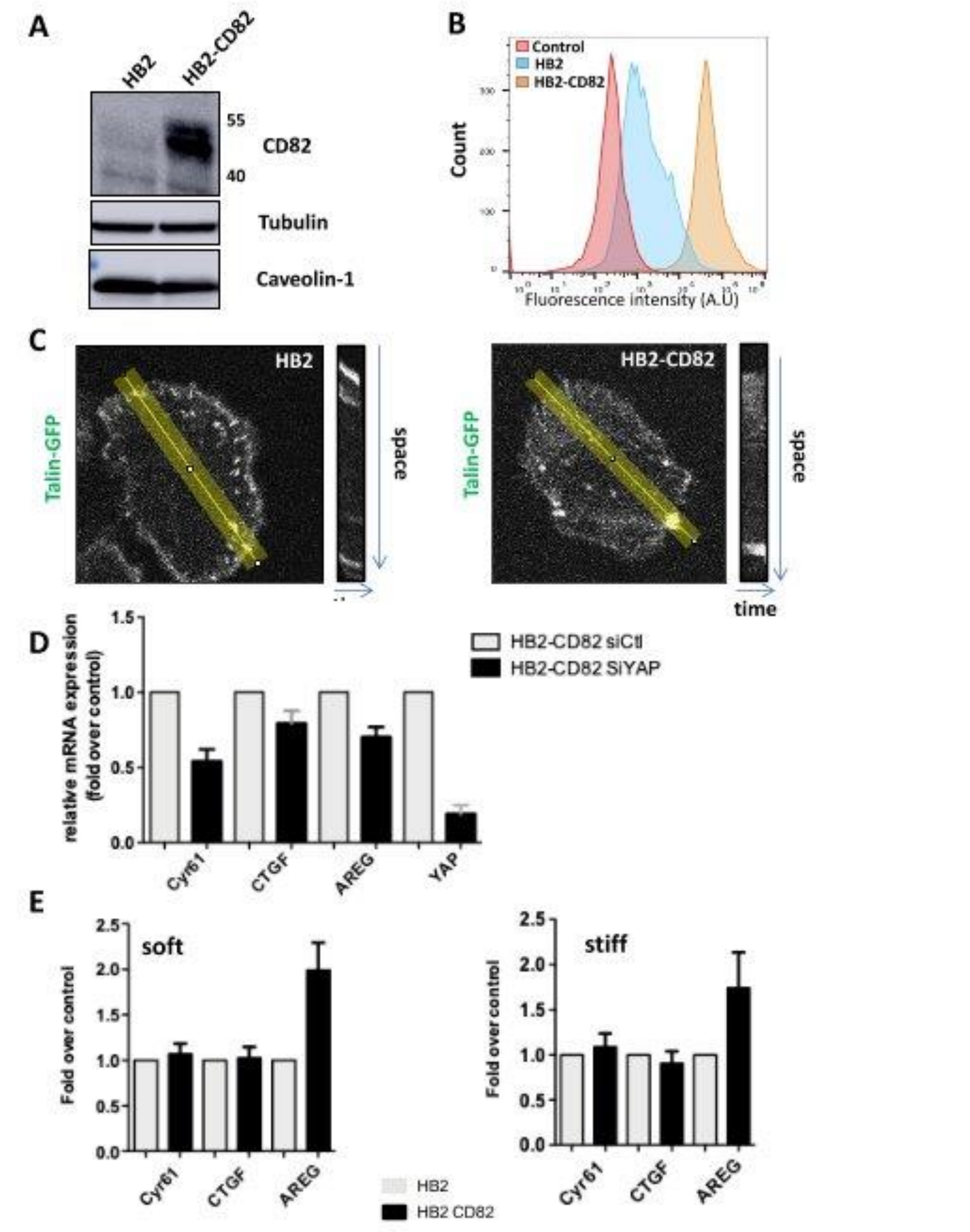

Figure S1: CD82 expression and its effect on talin-GFP, actin and YAP transcriptional targets in HB2 cells.

Figure S1. CD82 expression and its effect on Talin-GFP, actin and YAP transcriptional targets in HB2 cells. A) CD82 and caveolin-1 expression in HB2 and HB2-CD82 cells by Western blot; tubulin is shown as a loading control. **B)** Cytometry analyses of CD82 expression in HB2 and HB2-CD82 cells, a control with only the secondary antibody is also shown. **C)** kymographs extracted from the supplementary Movies 3&4 for HB2 (left) and HB2-CD82 (right) cells; notice the cycling bands in HB2 cells (left) indicating Talin-GFP movements**. D**) RT-QPCR analysis of YAP targets genes in HB2-CD82 cells. Cells grown on stiff and transfected with Si ctl or siYAP showing YAP targets (mostly Cyr 61 and AREG) in HB2-CD82 cells **E)** HB2 cells grown on soft and stiff substrates (one representative experiment carried out in triplicate over 3 independent experiments) showing AREG mRNA overexpression in CD82 overexpressing cells.

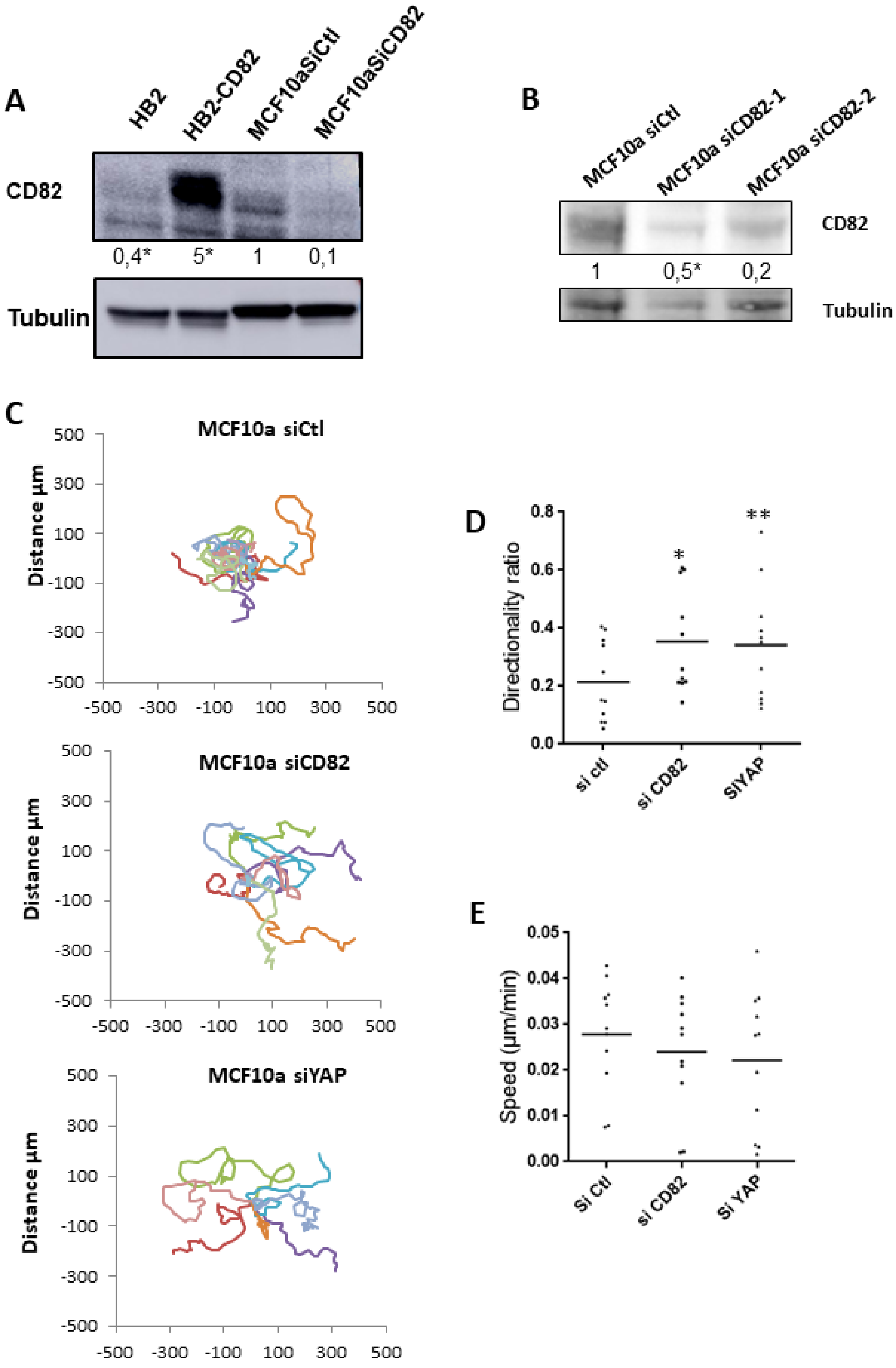

Figure S2: CD82 expression and its effect on EGF-induced cell migration in MCF10a

Figure S2. CD82 expression and its effect on EGF-induced cell migration in MCF10a. A) CD82 and caveolin-1 expression in MCF10a cells and HB2 cells by Western blot; tubulin was used as a loading control and the numbers are from gel quantification performed with Image J software. * indicates that values have been corrected for the amount of tubulin; **B)** CD82 expression following downregulation by SiRNA from Santa Cruz (siCD82-1) and Eurogentec (SiCD82-2). Tubulin was used as a loading control. The numbers are from gel quantification preformed with image J software, **indicates that values were corrected for the amount of tubulin; **C)**Trajectories of EGF-stimulated MCF10a cells transfected with siCtl, SiCD82 or siYAP (for clarity only 10 trajectories are shown per condition); **D)** Directionality ratio of the cells determined as the shortest distance between the starting and arrival points divided by the real distance traveled by the cells; **E)** Average speed of the cells, n= 11, respectively; *$p<0.05$

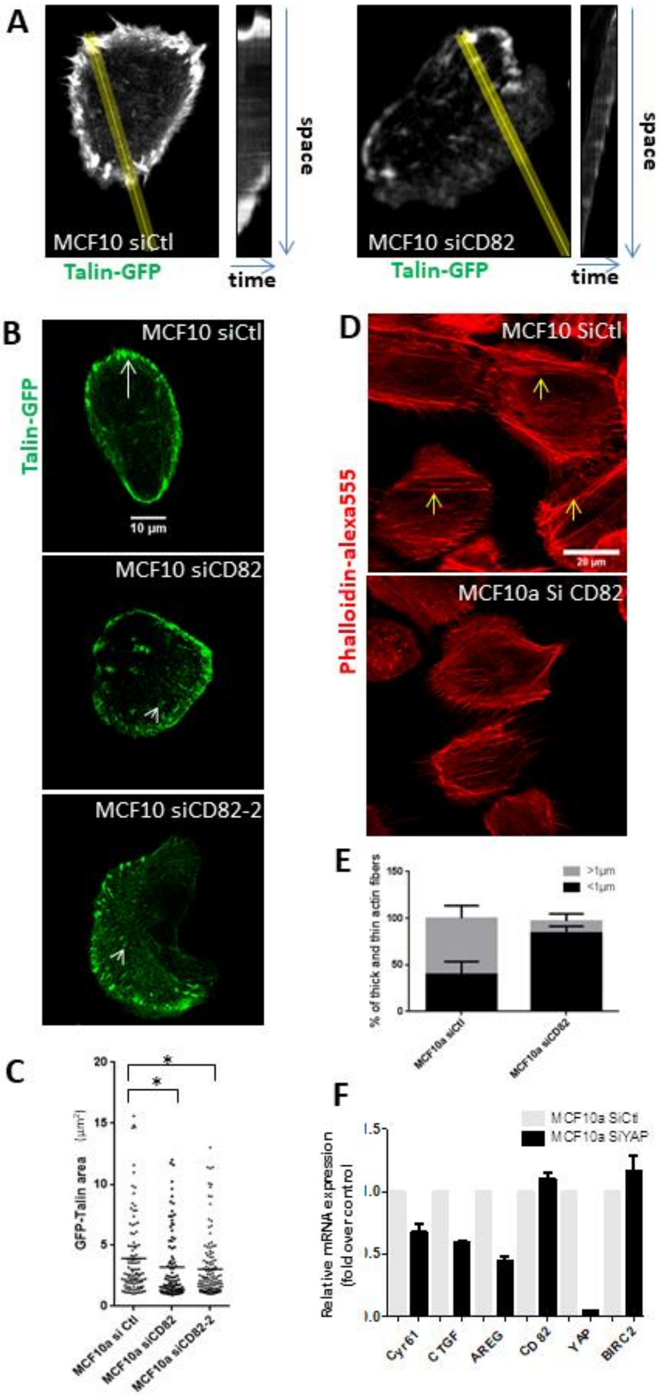

Figure S3: CD82 expression and its effect on talin-GFP , actin and YAP transcriptional targets in MCF10a

Figure S3. CD82 expression and its effect on talin-GFP, actin and YAP transcriptional targets in MCF10a. A) kymographs extracted from supplementary Movies 5&6 for MCF10a cells transfected with SiRNA control or SiRNA targeting CD82. Notice the cycling bands indicating Talin-GFP movements in MCF10 Si CD82. **B**) Talin-GFP labelling in MCF10a siCtl (left panel) and MCF10a siCD82-1 and siCD82-2 (SiRNA validation shown in Figure S2B). **C)** Quantification of the size of the peripheral adhesions on 11 cells from 3 independent experiments *$p<0.05$**.** **D**) Phalloidin-Alexa-555 labelling of actin in MCF10a siCtl and Si CD82cells (right panel). Arrows point out thick fibers in MCF10a siCtl; **E)** Quantification of the fibers thickness shown in D using Fiji line plot plugin (measurement of the width of the peaks), shown as the percentage of the total number of fibers on 20 cells from 4 independent experiments. **F)** RT-QPCR analysis of YAP target genes in MCF10a SiCtl or SiYAP (one representative experiment in triplicate over 3 independent experiments). Notice the strong downregulation of YAP and the downregulation of the YAP targets Cyr61, CTGF and AREG.

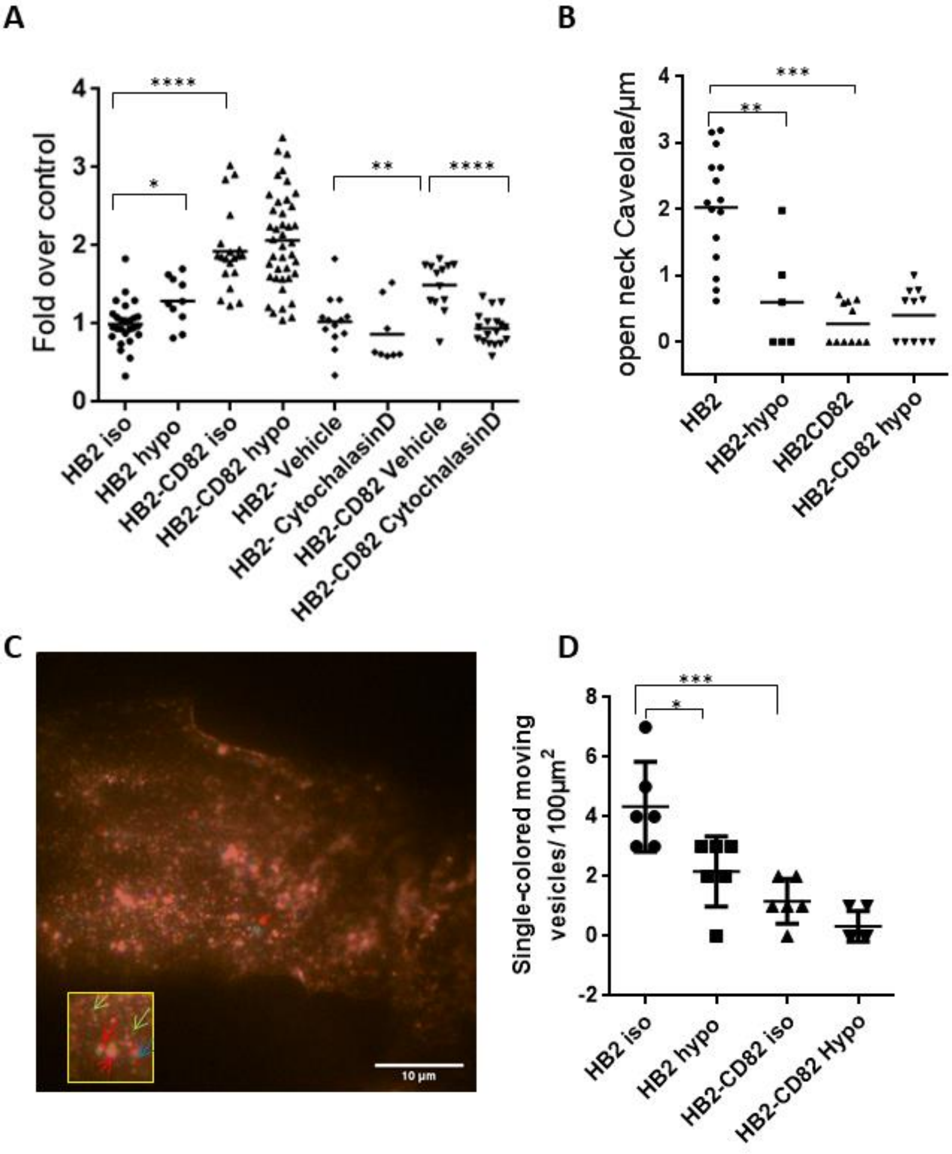

Figure S4: Regulation of membrane tension and cell surface caveolae density in HB2 cells

Figure S4. Regulation of membrane tension and cell surface caveolae density in HB2 cells A) membrane tension regulation by a mild hypoosmotic shock and upon cytoskeleton disruption. 3 cells were first analyzed then water (hypoosmotic shock) or 200µg/ml cytochalasin D or vehicle (DMSO) was added to the plate, incubated for 5 min and 3 additional cells were analyzed. * $p<0.05$, ****$p<0.0001$, ns non-significant. **B)** Quantification of the number of caveolae per µm of plasma membrane in HB2 and HB2-CD82 cells under iso or mild hypotonic conditions. **$P<0.01$, ns: non-significant**.** **C)** Example of image quantification: 3 successive images were extracted from Movie 9 (TIRF imaging of caveolin 1-GFP in HB2 cells under isotonic conditions). They were taken 1 sec apart and colored blue, green and red, respectively and then merged (image shown). Vesicles moving in the 1 sec time frame appeared monochromatic while vesicles not moving have merged colors (yellow, violet, white). The insert highlights monochromatic moving vesicles. D) Quantification of moving vesicles in 3 different areas of $100\mu m^2$ from 2 movies of HB2 and HB2-CD82 cells under iso or hypotonic conditions. *$p<0.05$, ***$p<0.001$.

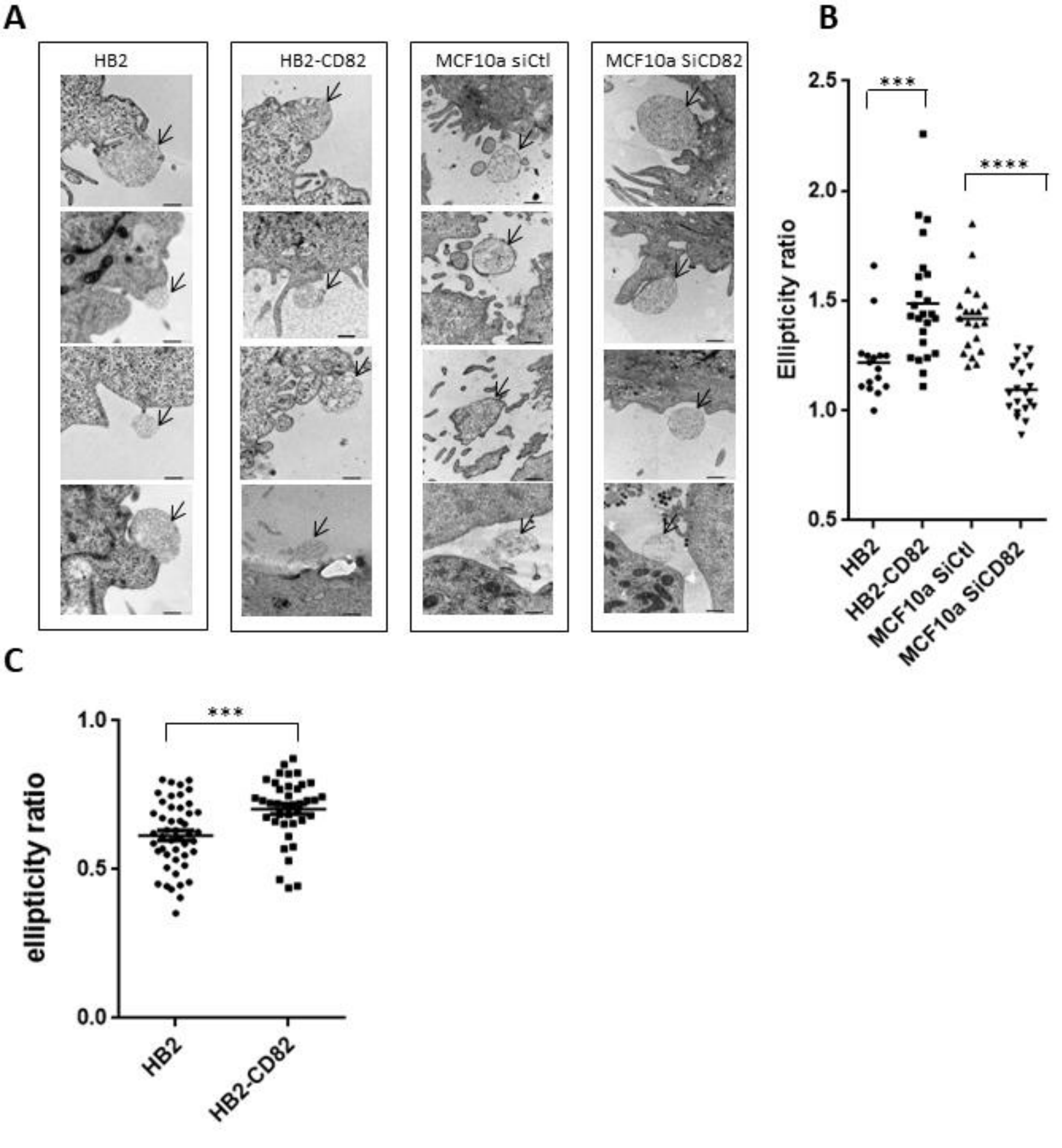

Figure S5: Migrasomes (A&B) and nuclei (C) deformation induced by CD82

Figure S5. Migrasomes and nuclei deformation induced by CD82. A) Panel of micrographs showing migrasomes (black arrows) in HB2, HB2-CD82, MCF10a cells silenced or not for CD82. B) Quantification CD82-induced deformation of migrasomes. The ellitipcity ratio was determined by dividing the long axis by the short axis of intact migrasomes in HB2, HB2-CD82, MCF10a siCtl and MCF10a SiCD82 from 16, 24, 20 and 20 cells, respectively. ***p<0.001, ****p<0.0001. A ratio of 1 indicates round migrasomes. C) The ellipticity ratio of nucleus was determined by dividing random measured axes in HB2 vs HB-CD82 cells plated on glass-coverslips (47 and 39 cells, n=3, respectively). A ratio of 1 indicates round nuclei (***p<0.001).
