# Supplementary figures and images for: Mechanical Control of Cell Migration by the Metastasis Suppressor Tetraspanin CD82/KAI1
Source: Cells. 2021 Jun 18;10(6):1545. doi: 10.3390/cells10061545 (PMC8234748; doi:10.3390/cells10061545)

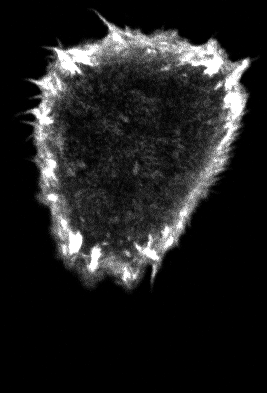

Supplement: Supplementary file 1 [file cells-10-01545-s001.zip › cells-1192922 suppl movies/Suppl Movie 7 MCF10a_siCTL__.tif]

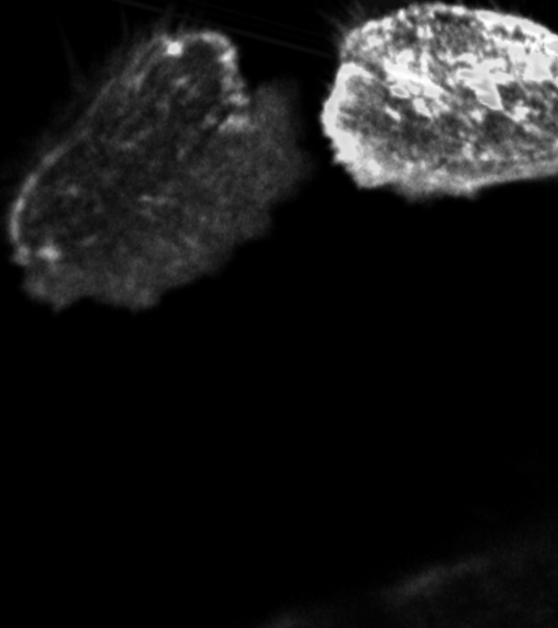

Supplement: Supplementary file 1 [file cells-10-01545-s001.zip › cells-1192922 suppl movies/Suppl movie 8 MCF10a_siCD82.tif]
